# Paraneoplastic Neuropathies: What's New Since the 2004 Recommended Diagnostic Criteria

**DOI:** 10.3389/fneur.2021.706169

**Published:** 2021-10-01

**Authors:** Marco Zoccarato, Wolfgang Grisold, Anna Grisold, Valentina Poretto, Federica Boso, Bruno Giometto

**Affiliations:** ^1^Neurology Unit O.S.A., Azienda Ospedale-Università di Padova, Padova, Italy; ^2^Ludwig Boltzmann Institute for Experimental and Clinical Traumatology Donaueschingenstraße 13 A-1200 Vienna, Vienna, Austria; ^3^Department of Neurology, Medical University Vienna, Vienna, Austria; ^4^Neurology Unit, Ospedale S Chiara, Azienda Provinciale per i Servizi Sanitari (APSS), Trento, Italy; ^5^Department of Neurology, University of Trieste, Trieste, Italy

**Keywords:** cancer, tumor type, paraneoplastic neuropathy, onconeural antibodies, surface antibodies, mechanisms, therapy

## Abstract

The diagnostic criteria published by the PNS (Paraneoplastic Neurological Syndromes) Euronetwork in 2004 provided a useful classification of PNS, including paraneoplastic neuropathies. Subacute sensory neuronopathy (SSN) was the most frequently observed peripheral PNS, whereas other forms of neuropathy, as sensory polyneuropathy, sensorimotor polyneuropathy, demyelinating neuropathies, autonomic neuropathies, and focal nerve or plexus lesions, were less frequent. At the time of publication, the main focus was on onconeural antibodies, but knowledge regarding the mechanisms has since expanded. The antibodies associated with PNS are commonly classified as onconeural (intracellular) and neuronal surface antibodies (NSAbs). Since 2004, the number of antibodies and the associated tumors has increased. Knowledge has grown on the mechanisms underlying the neuropathies observed in lymphoma, paraproteinemia, and multiple myeloma. Moreover, other unrevealed mechanisms underpin sensorimotor neuropathies and late-stage neuropathies, where patients in advanced stages of cancer—often associated with weight loss—experience some mild sensorimotor neuropathy, without concomitant use of neurotoxic drugs. The spectrum of paraneoplastic neuropathies has increased to encompass motor neuropathies, small fiber neuropathies, and autonomic and nerve hyperexcitability syndromes. In addition, also focal neuropathies, as cranial nerves, plexopathies, and mononeuropathies, are considered in some cases to be of paraneoplastic origin. A key differential diagnosis for paraneoplastic neuropathy, during the course of cancer disease (the rare occurrence of a PNS), is chemotherapy-induced peripheral neuropathy (CIPN). Today, novel complications that also involve the peripheral nervous system are emerging from novel anti-cancer therapies, as targeted and immune checkpoint inhibitor (ICH) treatment. Therapeutic options are categorized into causal and symptomatic. Causal treatments anecdotally mention tumor removal. Immunomodulation is sometimes performed for immune-mediated conditions but is still far from constituting evidence. Symptomatic treatment must always be considered, consisting of both drug therapy (e.g., pain) and attempts to treat disability and neuropathic pain.

## Introduction

The paper published in 2004 by Graus et al. (1) on behalf of the PNS (Paraneoplastic Neurological Syndromes) Euronetwork provided a useful classification of PNS, including neuropathies. At that time, subacute sensory neuronopathy (SSN) was the most frequently observed neuropathy (“classical” paraneoplastic neuropathy) associated with cancer, while other entities, as sensory polyneuropathy, sensorimotor polyneuropathy, and demyelinating neuropathies, were less frequent. The other classical peripheral syndrome defined in 2004 was chronic gastrointestinal pseudo-obstruction. Several subsequent works have reported novel antibody associations, further types of tumor associations, and different types of neuropathy, several of which were not contained in the former classification. A recent update of the classification (2) confirmed the range of clinical presentations of neurological syndromes typically associated with cancer (“high-risk neurologic phenotypes”), including SSN. In this review we aim to provide an update on mechanisms, antibodies, clinical presentation, and management of paraneoplastic neuropathies focusing on the pathological entities.

## Mechanisms

The mechanisms underlying paraneoplastic neuropathies (PN) are manifold and not uniform for individual neuropathies or tumor types. Although paraneoplastic neuropathies have been known for a long time, the autoimmune hypothesis only appeared in 1965 when Wilkinson and Zeromski described antibodies against neurons in paraneoplastic sensory neuropathy for the first time (3). At the core of the autoimmune hypothesis is the production of antibodies against neural antigens determined by an immune response to cancer.

The main focus of the 2004 classification was on onconeural antibodies, targeting intracellular antigens shared by neuronal and tumoral tissues. The pathogenic role of onconeural antibodies remains unresolved. The most widely recognized hypothesis is that T-cell cytotoxicity accounts for neuronal cell loss in these conditions (4). Today, however, the mechanisms causing paraneoplastic neuropathies by far exceed the spectrum of onconeural antibodies (Table 1). Some peripheral conditions, e.g., peripheral nerve hyperexcitability syndromes (5), are mediated by neuronal surface antibodies (NSAbs). The term “surface antibodies” indicates antibodies targeting antigens present on the membrane of neurons, thus producing a potential direct effect, such as ion channel dysfunction.

**Table 1:** Level of characterization and frequency of mechanisms underlying paraneoplastic neuropathies.

**Associations/mechanisms**	**Level of characterization**	**Frequency in the paraneoplastic neuropathy spectrum**
Onconeural abs	+ + +	+ + +
NSAbs	+ + +	+
Other immune-mediated mechanisms	–	–
Hematologic diseases, including amyloid	++	++
Weight loss, cachexia, infection	–	+ + +

*NSAbs, Neuronal Surface Antibodies*.

Immune-mediated mechanisms determine also the rare presentations of Guillain-Barré syndrome (GBS), chronic inflammatory demyelinating polyneuropathy (CIDP), and vasculitis. Reference is occasionally made to these large groups of diseases in a paraneoplastic context. The exact trigger and relationship between the immune response and cancer is not clear, but it is doubtful that the same mechanisms apply to all these entities.

Plasma cell dyscrasia and other hematological entities present with a spectrum of different neuropathies of both axonal and demyelinating forms, like anti-myelin-associated glycoprotein (MAG) neuropathy, POEMS (polyneuropathy, organomegaly, endocrinopathy, monoclonal-protein and skin changes) syndrome, and immunoglobulin light-chain (AL) amyloidosis. MAG and other antibodies targeting myelin-associated glycoprotein and glycolipids are deemed to be pathogenic (6). Hyperproduction of light chains could have a direct toxic effect but overall leads to the formation of amyloid deposits with extracellular accumulation of fibrils and consequent axonal damage. In addition, hyperviscosity effects have been claimed in Bing-Neel syndrome with peripheral involvement in Waldendström's macroglobulinemia (7).

A number of causes for paraneoplastic sensorimotor neuropathies, especially in advanced stages of cancer, remain obscure and resemble the development of neuropathies in some general diseases, as infections and critically ill conditions (See the paragraph on “Sensory neuromyopathy and terminal neuropathy”).

By enhancing antitumor immunity, the emerging immune therapies, particularly immune checkpoint inhibitors (ICI), have been associated with a spectrum of immune-mediated diseases, including neuropathies and rapidly progressive polyradiculoneuropathies (8, 9).

## Antibody and Tumor Association

Several antibodies targeting neural antigens have been described in patients with PN, although this condition is well-known to occur also without antibodies (1, 10). The 2004 diagnostic criteria established that onconeural antibodies were consistently associated with PNS. Still they are now a fundamental marker supporting diagnosis of paraneoplastic neuropathy, commonly detected using commercial and in-house tests on cerebellar tissue with specific patterns and recombinant protein-based dot/line blotting (11). In 2004, onconeural antibodies were classified as “well-characterized” or “partially characterized” antibodies. This distinction was based on clinical relevance, number of published cases, a recognizable pattern on immunohistochemistry, availability of immunoblotting confirmation, and absence/low frequency in patients without cancer. The 2021 update of diagnostic criteria (2) classifies the antibodies associated with paraneoplastic diseases in three groups according to the frequency of association with cancer (high- >70%, intermediate-, and low- <30% risk antibodies), but in this review, we use the former nomenclature.

Well-characterized antibodies associated with paraneoplastic neuropathies included anti-Hu (ANNA-1), anti-CV2 (CRMP5), and anti-amphiphysin antibodies. Since 2004 the literature has confirmed that these three markers are consistently associated with the PN spectrum (12–19) (Table 2). SSN with anti-Hu antibodies is considered the most frequent PN (10). It is often the predominant manifestation of anti-Hu multifocal encephalomyelitis (paraneoplastic encephalomyelitis), which in 10% cases can also manifest with dysautonomic symptoms, as hypotension, dysrhythmia, or intestinal pseudo-obstruction (20). Two extensive case series have compared anti-Hu with anti-CV2 neuropathy (12, 13). Anti-CV2 neuropathy seems to be characterized more frequently by sensorimotor involvement and by asymmetric polyradicular involvement. SCLC has been confirmed as the most frequent cancer associated with both anti-Hu and anti-CV2 antibodies. Anti-amphiphysin antibodies are rare and associated with a broad spectrum of neurological manifestations, especially in women with breast cancer or SCLC. Peripheral involvement includes sensory neuronopathy, sensorimotor neuropathy, polyradiculopathy, and neuromyotonia (14, 15).

**Table 2:** Onconeural antibodies reported in 2004 classification and associated with paraneoplastic neuropathy.

**Antibody (antigen)**	**Types of neuropathy**	**Associated tumors**	**Selected published series after 2004 criteria**
Hu (ANNA1)	SSN; autonomic neuropathy	SCLC	(12, 13, 17–19)
CV2 (CRMP5)	Sensorimotor neuropathy	SCLC; thymoma	(12, 13)
Amphiphysin	Polyradiculoneuropathy, SSN	Breast, SCLC	(14, 15)
ANNA3	Sensorimotor neuropathy	SCLC	/
PCA-2/MAP1B	Sensorimotor neuropathy; autonomic neuropathy	SCLC	(16)

Partially characterized antibodies in 2004 included ANNA-3, a very rare reactivity reported in some cases of sensory, sensorimotor and autonomic neuropathy (21), anti-Zic4—a marker of cerebellar degeneration described in some cases of neuropathy, usually with concomitant antibodies (anti-Hu and/or anti-CV2) (22)—and anti-PCA2, which in the earlier large case series (23) was described in both central and peripheral syndromes, including LEMS and neuropathy. The real target of PCA-2 was identified in 2017, consisting of a microtubule-associated protein (MAP1B). PN is the most frequent presentation and was reported in about a half of anti-MAP1B patients with SCLC (16).

In the last 15 years, several novel onconeural antibodies related to PN have been reported. Most have been described in individual works, have not yet been confirmed by other research groups, and have a limited number of patients. The strength of these associations thus needs further characterization and confirmation before being considered relevant in the diagnostic approach to PN (Table 3).

**Table 3:** Novel antibodies associated with paraneoplastic neuropathy.

**Antibody (antigen)**	**Localization (intracellular/surface)**	**Types of neuropathy**	**Associated tumors**
AGNA/SOX1	Intracellular	Sensory neuronopathy, sensorimotor neuropathy	SCLC
ITPR1	Intracellular	Sensorimotor polyneuropathy	Lung, Multiple Myeloma
KLHL 11	Intracellular	Myeloneuropathy	Testicular seminoma
LZUP4 (Leucin-Zipper 4)	Intracellular	Motor neuropathy, polyradiculopathy	Germ cell tumors
NfL (Neurofilament light chain)	Intracellular	Peripheral and cranial neuropathy	Neuroendocrine lineage neoplasms
CASPR2	Surface	Neuromyotonia, painful neuropathy, Morvan syndrome	Thymoma (20–30%)
LGI1	Surface	Neuromyotonia, painful neuropathy	Thymoma (5–10%)
Netrin 1 receptors	Surface	Neuromyotonia	Thymoma
		Morvan syndrome	

In 2016, antibodies against 1,4,5-trisphosphate receptor type 1 (ITPR1) were characterized in patients with autoimmune cerebellar ataxia; this antibody was also found in three patients with sensorimotor polyneuropathy, associated in two cases with malignancy (adenocarcinoma of the lung and multiple myeloma) (24).

Antibodies targeting neurofilament light chain (NfL) have been reported as markers of ataxia and encephalopathy accompanying various cancers, especially neuroendocrine lineage neoplasms (25). Six out of 21 of the reported patients manifested with peripheral or cranial neuropathy.

Anti-KLHL11 is a recently described antibody identified in patients with brainstem encephalitis or cerebellar symptoms related to testicular and ovarian cancer (26, 27). A recent retrospective study of 32 patients manifesting a distinctive pattern of subacute paraneoplastic myeloneuropathy, associated with several neoplasms, identified the presence of anti-amphiphysin (8), anti-Hu (5), anti-CV2 (6), anti-Yo (1), anti-PCA2 (2), and one case of anti-KLHL11, or combinations of these (28).

Very recently, a further antibody reaction against Leucine Zipper 4 (LZUP4) was described in 28 patients with germ cell tumors (e.g., seminoma); four patients were affected with motor neuronopathy and polyradiculopathy (29).

In this context, the characterization of antibodies against Sry-like high mobility group box 1 (SOX1) deserves mention. In 2005, anti-glial nuclear antibody (AGNA) was identified as a marker of PNS-related lung cancer. The most frequent clinical association was LEMS, but SSN and sensorimotor neuropathy were also reported (30, 31). SOX1 was identified as the antigen in 2008 (32). As anti-SOX1 antibody is not a rare finding in patients with SCLC without paraneoplastic accompaniments (33), it can more properly be considered a serological marker of SCLC. Recently, the presence of SOX-1 was also advocated in non-paraneoplastic neuropathies (34, 35), but this finding was not confirmed in a later work (36).

In recent years several antibodies directed against NsAbs have been described, greatly expanding the interest in autoimmune neurological diseases. Antibodies against voltage-gated potassium channels (VGKC) were first discovered in the 1990s in patients with motor nerve hyperexcitability (neuromyotonia) and hyperhidrosis (37, 38). Anti-VGKC antibodies were later described in limbic encephalitis, with most cases being of non-paraneoplastic origin (39, 40). In 2010, it was established that anti-VGKC antibodies actually target two different associated proteins associated with ion channels, namely, LGI1 and CASPR2 (41, 42). These antibodies determine a continuous disease spectrum, often dominated by central nervous system involvement (limbic encephalitis) but frequently with relevant peripheral manifestations, including neuromyotonia, dysautonomia, and pain (43). Peripheral involvement, especially when isolated, is more frequent in CASPR2 than in LGI1 patients; this is probably due to the higher expression of CASPR2 in the peripheral nervous system, in the juxta-paranodal region (44). Morvan syndrome is another clinical picture associated with CASPR2 antibodies, in which peripheral hyperexcitability and dysautonomia coexist with psychiatric disturbances and sleep dysfunction. Recently, painful manifestations of LGI1 and CASPR2 autoimmunity have been highlighted. Sometimes pain is the cardinal symptom, especially in CASPR2 patients (45–47). As regards the associated tumors, anti-LGI1 diseases are rarely paraneoplastic, whereas a tumor, mostly a thymoma, can be detected in 20–30% of patients with CASPR2 autoimmunity. In patients with double positivity (anti-LGI1/anti-CASPR2), the likelihood of cancer is higher (up to 44%) (47).

Finally, a further surface reaction against Netrin-1 receptor has recently been described in patients with thymoma affected by Morvan syndrome or neuromyotonia and myasthenia gravis; the clinical relevance of this reactivity, which can be found together with CASPR2, needs further specification (48, 49).

Tumor association reflects antibody association (see Tables 2, 3). The most frequent malignancy associated with PN is SCLC. However, other lung cancers, like adenocarcinoma, are not rare. Other associated malignancies are breast and ovarian cancer, and thymoma. Prostate cancer is an infrequent finding in the neurological paraneoplastic context, considering the frequency of the neoplasm, although cases of SSN have been reported with Hu antibodies (50). When the diagnosis of PNS is performed in a patient with an unknown or occult malignancy, oncological screening must be performed as recommended (51).

## Types of Neuropathies

Over the decades, numerous individual observations have been published. Gradually, a core of generalized neuropathies has emerged, in particular, SSN. Several rarer types need to be considered, as do focal peripheral nerve lesions. The spectrum is probably still incomplete but reflects the present level of knowledge and experience (Table 4).

**Table 4:** Classification of paraneoplastic neuropathies.

**Types of PN neuropathies**	**Sub-classification**	**Comments**
Generalized neuropathies	Subacute sensory neuronopathy	Highly indicative of a PN cause- although also observed in other conditions
	Sensory neuropathy	Unspecific
	Sensorimotor neuropathy	Unspecific
	Sensory neuromyopathy and terminal neuropathy	Historic terminology
	Motor neuropathy	Rare
	Multiplex neuropathy	Vasculitis is rare
	Myeloneuropathy	Possibly new entity
	Autonomic neuropathies	Incidence uncertain
Rarer and disputed entities	Small fiber neuropathy	Incidence unclear, except for hematologically-associated types
	Cryoglobulinemic neuropathy	
	Hyperexcitability syndromes	
	Paraproteinemia and AL amyloid	
Focal nerve lesions	Cranial nerves	Individual cases
	Nerve plexus	
	Mononeuropathies	

### Generalized Neuropathies

#### Subacute Sensory Neuronopathy

SSN is the prototype of PN. It is characterized by asymmetry, acute/subacute onset in the upper extremities, and pain. The main clinical feature is sensory ataxia. Even though the motor system is not impaired in terms of strength, sensory loss results in ataxia and severe disability. The disease quickly progresses to a plateau phase, and has little or no tendency to improve. Electrophysiologic study reveals absent sensory responses; motor responses can be minimally altered (52). The neuropathology consists of inflammation in the dorsal root ganglia (DRG) and is often associated with posterior column degeneration. The therapeutic approaches are manifold (53) and usually immune modulation is recommended, although no evidence-based recommendations exist. SSN is a disabling condition and persons affected remain completely dependent. SSN is not entirely specific and has been observed as an idiopathic occurrence or in other autoimmune conditions as Sjogren's syndrome. The DRG can be affected by toxicity, as through platinum compounds and pyridoxine overdosage, but the extent of the clinical symptoms is not the same as in Hu-associated paraneoplastic SSN. SSN has also been observed in association with other PNS as cerebellar degeneration, PEM, brainstem involvement, limbic encephalitis, Lambert–Eaton myasthenic syndrome, and motor neuropathy. Patients may have evidence of autonomic involvement. A rare combination with myositis has been described (54). For the clinician, the appearance of an SSN constitutes a strong recommendation to search for cancer, particularly SCLC.

#### Sensory Neuropathy

A pure sensory neuropathy is less specific for a paraneoplastic disease, and opinions regarding paraneoplastic etiology are controversial. Patients present with sensory symptoms in the typical glove-stocking distribution, often with neuropathic pain and a variable degree of incoordination (55). Onset is usually more insidious, following the length-dependent distribution. The most likely causes of a pure sensory neuropathy are autoimmune diseases (e.g., Sjögren) and toxic and metabolic neuropathies. The distinction between SSN and sensory neuropathy can be difficult; even in the 2004 classification it was not always certain how precise the distinction could be. Subacute or acute onset may resemble SSN. An attempt to differentiate ataxic from more painful sensory neuropathies was made by Oki et al. (18) and may help in differentiation.

#### Sensorimotor Neuropathy

Sensorimotor neuropathy (SMN) is generally the most frequent yet enigmatic neuropathy in terms of specific characteristics and etiology. The term implies a combination of sensory and motor symptoms of varying degrees. No specific or characteristic clinical items have been defined. Patients with SMN usually do not have severe neurological impairment. SMN has been observed as a paraneoplastic condition, also in association with onconeural antibodies (55, 56). The differential diagnosis is wide and ranges from other causes as alcohol, diabetes, and chronic idiopathic axonal polyneuropathy, particularly in individuals aged over 55 years (57).

Yet individual cases with SMN presenting as the first sign of cancer have been described. Practically speaking, SMN is uncharacteristic and the suggestion is to first exclude other possibilities before embarking on an extensive tumor search. At a later stage of the cancer, chemotherapy-induced peripheral neuropathy (CIPN) is the most likely differential diagnosis. In plasma cell dyscrasia and associated hematological diseases, a spectrum of neuropathies has been described, including SMN (see below).

#### Sensory Neuromyopathy and Terminal Neuropathy

Paraneoplastic sensory neuromyopathy is usually a late effect of cancer on the peripheral nervous system. It presents as a symmetric sensorimotor neuropathy, is usually mild, and can be associated with type 2 fiber muscle atrophy. It is not specific for individual cancers, is often associated with weight loss, and can be a general sign of advanced cancer. The sensory loss affects all qualities. Muscle weakness occurs in proximal and distal muscles (with so-called “intermediate sparing”). It is slowly progressing. Concomitant factors as chemotherapy or diabetes need to be ruled out. There is no specific antibody association at present. The nomenclature is historic and was not contained in the 2004 classification. However, in clinical practice, this type of neuropathy can be observed in advanced cancer patients.

The term “terminal neuropathy” refers to mild sensorimotor neuropathies observed in progressive general diseases, in advanced stages, including cancer, with or without concomitant use of neurotoxic drugs. This type of neuropathy can be likened to a common occurrence in patients with severe infections, i.e., weight loss, and its origin is probably different. For example, sarcopenia and cachectic neuropathy have been described in association with diabetic neuropathy (58, 59).

#### Motor Neuropathy

Pure motor neuropathy is rare. The term “lower motor neuron disease” has been used to identify patients with subacute development of generalized flaccid paresis with sparing of long tracts and bulbar muscles (60). This entity has been described infrequently and is not well-characterized. Only a few cases were collected in the PNS Euronetwork database (61), corresponding to <1% of identified patients. The term “lower motor neuropathy” has been also used in conjunction with plasma cell dyscrasia and myeloma (62, 63), in hematological malignancies, and as a sequelae of local RT.

#### Multiplex Neuropathy

Despite being described in individual cases, paraneoplastic mononeuropathy multiplex is a rarePNS (64, 65). Mononeuropathy multiplex is generally associated with vasculitis. Chronic dysimmune neuropathies, as multifocal motor neuropathy (MMN), have been reported as PN (66), while rare cases of asymmetric neuropathies have been observed in AL amyloidosis (67) and in association with Waldenstrom's disease (68). Recently observed complications of ICI therapies also include reports of vasculitis (69). Notably, also other tissues can be involved by vasculitis as a clinical sign, e.g., skin vasculitis (70) and digital ischemia (71).

#### Myeloneuropathy

The simultaneous involvement of the spinal cord and peripheral nerves is termed myeloneuropathy. The usual underlying etiologies include B12 or copper deficiency, inflammatory/infectious diseases, and toxic diseases. This is a fairly new term in conjunction with PNS, which is not contained in classical descriptions. A recent paper (28) has described a number of cases that could be the basis for further considering its inclusion in the PNS classification. The series consists of 34 patients with various antibodies and cancers, especially SCLC and breast adenocarcinoma. Clinically they had a subacute, asymmetric presentation with sensory (e.g., paresthesias and impaired proprioception) and motor signs, as asymmetric weakness, and long tract signs. Pain and bladder dysfunction were frequent clinical findings. The concomitant presence of hyporeflexia and hyperreflexia at different sites was found in 81% of patients. MRI imaging showed spinal hyperintensity, which in about a half of patients was longitudinally extended. One third of patients showed lumbar nerve-root enhancement. Previous descriptions of similar syndromes are available (72, 73).

#### Autonomic Neuropathy

Autonomic features can be associated with several types of neuropathy. Symptoms can result in orthostatic hypotension, urinary symptoms, sweating abnormalities, intestinal pseudo-obstruction, gastrointestinal dysmotility, and subacute dysautonomia. Dysautonomia is typical of AL amyloid neuropathy. It has likewise been observed in several paraneoplastic forms of PN (74) and small fiber neuropathy (75). Another important aspect is the association of autonomic symptoms with other PNS, in particular with LEMS, but also in conjunction with paraneoplastic hyperexcitability syndromes as Morvan syndrome. In addition to areflexia, stocking-like sensory loss, and areflexia, autonomic symptoms also appear. Autonomic ganglionopathies have been observed in conjunction with ICI therapy (76). Notably, autonomic disturbances are also well-described in other common causes of neuropathy, as diabetes or neuropathies associated with systemic autoimmune diseases.

### Rarer and Disputed Entities

#### Small Fiber Neuropathy

Small fiber sensory neuropathy (SFN) has not been part of the classical paraneoplastic spectrum. A recent study suggests that paraneoplastic causes can amount to 3% of SFN in association with onconeural antibodies that were not further specified (77). SFN has been documented in cases of Morvan syndrome (78). Although examples of SF involvement have been described in PN in solid cancers (79) and hematological diseases (80), it remains unclear whether the SF involvement is exclusive, or part of a general neuropathy syndrome.

#### Immune-Mediated Neuropathies

From the large, growing number of immune-mediated neuropathies, GBS, CIDP, and also rarely MMN have been mentioned to appear as a paraneoplastic phenomenon. In the PNS Euronetwork database, the incidence of this conjunction was low. The occurrence of both GBS and CIDP seems higher in hematological conditions (81, 82) and has also been described to be associated with other cancer types (74, 83) and with axonal loss. The occurrence of MMN as a PNS has been reported but seems to be extremely rare (84, 85).

Practically speaking, the appearance of either GBS or CIDP does not necessarily point to an underlying malignancy. Conversely, GBS may occur during the course of the disease in cancer patients with solid tumors (86) but can be the presenting phenotype in lymphoma or leukemia, and may be called “paraneoplastic” in these circumstances.

As a new development and based on immune mechanisms, several immune-mediated neuropathies, also resembling GBS, have been described to occur in conjunction with immune therapies, in particular with ICIs (9, 87).

#### Cryoglobulinemia

Cryoglobulinemia can occur as part of lymphoproliferative disease as in MGUS, macroglobulinemia, multiple myeloma (MM), leukemia, CLL, and immunoblastic lymphadenopathy. In addition to acrocyanosis, digital necrosis and purpura may occur. Likewise, signs of hyperviscosity, as thrombosis, can be associated. Cryoglobulins are monoclonal IgG, often IgM, and rarely light chain. Cryoglobulinemic neuropathy has been described as paraneoplastic, with IgM precipitation (88) and other conditions. Except for lymphoproliferative diseases, where the coexistence has been noted, it does not seem to be a frequent association.

#### Hyperexcitability Syndromes

Abnormal muscular activity with cramps, twitching and stiffness characterizes neuromyotonia and hyperexcitability syndromes. Electromyographic findings are typical (89). Acquired forms are considered immune mediated and can be found with underlying neoplasms, including in seronegative cases. Neuromyotonia can also present with symptoms and signs of sensorimotor and motor neuropathy, with no clear mechanisms (90). Neuropathic pain is a frequent finding in Morvan syndrome (see above) (91).

#### Plasma Cell Dyscrasia and Paraproteinemia

Plasma cell dyscrasias and paraproteinemias are usually not classified among paraneoplastic neuropathies. This is probably due to the inhomogeneous presentations within the different groups with paraproteinemia. Despite this, several types of neuropathies occur in association with the hematological disease by indirect involvement (not directly neoplastic), resembling the pattern of paraneoplastic disease. At least three conditions, such as POEMS syndrome, anti-MAG neuropathy, and AL amyloidosis, have characteristics of paraneoplastic disorders. The mechanisms are diverse and range from immunoglobulin (IgG) deposition to axonal and demyelinating neuropathies, hyperviscosity issues, and AL amyloid deposition.

Neuropathies related to MGUS are usually sensory-motor, axonal, or demyelinating. Commonly, IgG or IgA cause axonal lesions, whereas IgM tends to be associated with demyelinating features.

Waldenstrom's disease can develop from MGUS and is considered as a low-grade lymphoma (lymphocytoplasmatic lymphoma). It can be associated with demyelinating neuropathies, sometimes with MAG antibodies. Hyperviscosity mechanisms can also result in a multifocal neuropathy (92). Cryoglobulinemia can be associated; AL amyloidosis is rare.

Multiple myeloma presents with a variety of PN, including axonal and demyelinating types with no typical clinical features. AL amyloidosis is responsible for 30–40% of neuropathies in myeloma, in particular with λ light chain.

Three phenotypes further illustrate the relationship between PNS and plasma cell dyscrasia. Anti-MAG neuropathy results in a painful sensory neuropathy, predominantly in the legs with impaired balance. Progression is slow and leads to sensory ataxia. Tremor action is frequently reported. Numerous investigations have discussed the effects of anti-MAG and the role of sulfoglucuronyl-glycosphingolipid (SGPG) antibodies (6). Therapy suggestions are controversial, among them anti-CD20 drugs, like rituximab, and attempt to neutralize the MAG antibodies (93).

POEMS syndrome is considered by many to be a PNS. The disease is often rapidly devastating, resulting in neuropathy with tetraparesis and multi-organ involvement. The neuropathy is M-protein related (Ig A or G) and can cause axonal, demyelinating, or mixed neuropathy (61). Often, an isolated osteosclerotic lesion can be detected. The distinction between CIDP and POEMS can be difficult and neurophysiological criteria have been suggested (94). A CIDP-like presentation with pain could be a useful indicator for POEMS. Elevated levels of vascular endothelial growth factor (VEGF) are found in about two-thirds of patients (95).

Light-chain (AL) amyloidosis is an important differential diagnosis in paraproteinemia, Waldenstrom's disease, multiple myeloma, and several other entities. Clinically, a combination of fatigue, renal impairment, sensory and autonomic neuropathy, and often also carpal tunnel syndrome (CTS) is suggestive. In addition to the characteristic sensory, often painful neuropathy, frequently including autonomic features, focal lesions due to deposits of amyloid are also described. Macroglossia, periorbital purpura, congestive heart failure, and orthostatic hypotension are other typical (but not specific) clinical findings (96) (Table 5).

**Table 5:** Neuropathies associated with hematological diseases.

**Hematological disease**	**Neurological syndrome**
MGUS	Anti-MAG neuropathy (^*^), POEMS
Waldenstrom‘s disease	Axonal, demyelinating, anti-MAG neuropathy (^*^), hyperviscosity syndromes
Multiple myeloma	Axonal, demyelinating, AL amyloid (^*^)
Lymphoma	Immune-mediated (^*^), rarely AL
Leukemia	Rarely AL amyloid in CLL (^*^) and hairy cell leukemia

* *Neuropathies which bear similarities with the characteristics of paraneoplastic syndromes*.

#### Focal Nerve Lesions

Focal paraneoplastic neurological syndromes are rare and usually not contained in the classifications. The list of entities provided here is incomplete and based on observations and case reports, lacking consistency and systematic approach. Focal presentations include cranial nerve lesions, plexopathies, mononeuropathies (e.g., CTS) (97), and also muscle involvement.

#### Cranial Neuropathies

There are numerous reports on paraneoplastic cranial nerve (CN) lesions (Table 6). Three CNs appear to be preferably affected: the optic, trigeminal, and vestibular nerves. In addition to lesions of the optic nerve (98, 99), also in combination with spinal cord lesions (100), there are several visual conditions, as cancer-associated retinopathy (101), melanoma-associated retinopathy (102), acquired night blindness, bilateral diffuse uveal melanotic proliferation, bilateral diffuse uveal melanotic proliferation, cone dystrophy and achromatopsia, and photophobia (103).

**Table 6:** Paraneoplastic involvement of cranial nerves.

**CN**	**Neoplastic**	**Paraneoplastic syndromes**	**Other mechanisms**
II	Base of the skull	Optic nerve neuropathy/neuritis	Other immune-mediated causes, hyper-viscosity
	Leptomeningeal carcinomatosis	CAR, MAR	
III, IV, VI	Base of the skull tumors/metastasis	Individual cases	/
	Orbital metastasis		
	Leptomeningeal carcinomatosis		
V	Local metastasis, base of the skull metastasis, mandibular metastasis	Numbness trigeminal nerve, or parts (chin, cheek)	Isolated facial pain, neuralgia
VIII	Leptomeningeal carcinomatosis	Few reports on vestibular neuritis	/
Caudal CNs	Base of the skull metastases	Not reported (isolated nerves)	Focal amyloid in plasma cell dyscrasia
	Leptomeningeal carcinomatosis		
	Lesions after the exit of CN of the bony skull		
Multiple CNs	Base of the skull metastasis	Few reports, not homogeneous	/
	Leptomeningeal carcinomatosis		

*CAR, cancer associated retinopathy; MAR, melanoma associated neuropathy*.

Oculomotor nerve lesions are rarely described as a PN. Local neoplastic causes and orbital myositis need to be excluded (104). However, extraocular muscle involvement has been described in AL amyloidosis, and local AL deposits in the lid may cause ptosis.

The trigeminal nerve can be affected in autoimmune and rheumatoid arthritis. Similarly, sensory trigeminal neuropathy, or the “numb chin” (105) and “numb cheek” (106) syndrome subtypes, have been reported to be caused by paraneoplastic mechanisms (107, 108). The presence of orofacial pain (109–111) and neuralgia have been suggested to be of paraneoplastic origin, among other causes. Vestibular damage and neuritis have been described as paraneoplastic phenomena in a few selected cases (112–114). There is a paucity of reports on possible damage to the caudal cranial nerves. Local amyloid depositions (115) in the soft palate and tongue (116) need to be considered under plasma cell dyscrasia. Several cases of possible multiple CN lesions, caused by a potential paraneoplastic mechanism, have been mentioned (117, 118). A similar distribution of affected CNs has been reported as a complication of treatment with immune checkpoint inhibitors (119) (see below).

#### Plexopathies

PN causing plexopathies are rare but have been reported in individual cases. Most reports date back some years, and new investigations, in particular with the aid of imaging techniques, could potentially detect a symptomatic cause. The cervical plexus is mentioned in regard to the phrenic nerve (120–122). Although there are reports that inflammatory paraneoplastic causes can affect the brachial and lumbosacral plexus, the evidence is based on individual case reports (123).

#### Mononeuropathies

In plasma cell dyscrasia, CTS could be a sign of amyloidosis. The issue of other isolated paraneoplastic mononeuropathies is uncertain. One report suggests a paraneoplastic ulnar nerve lesion (124); there is also mention of a peroneal nerve lesion (125). Multiple enlarged nerves (126) have been described in ultrasound as a PNS, but may lack a clinical correlate. Deposition of light chains causing individual nerve lesions has been described (97). In summary, the appearance of a mononeuropathy of paraneoplastic origin is not likely, except in the case of CTS associated with AL amyloid.

## Cancer Therapy in Differential Diagnosis

CIPN is the commonest form of neurotoxicity in cancer patients, causing a chronic neuropathy in about 30% and milder/temporary symptoms in up to 70% of those undergoing traditional chemotherapy (127). In addition to the burden on patients' quality of life, CIPN can lead to modifications or discontinuation of oncologic therapy, thus impacting on their overall prognosis. Even after cessation of chemotherapy, symptoms impacting the quality of life persist in half of patients (128). Alteration of cancer treatment due to CIPN involves 10–65% of patients; in up to one third of patients, chemotherapy needs to be discontinued (129).

Despite significant variability in susceptibility [depending on concurrent diabetes, alcohol consumption, and other pre-existing neuropathies, along with genetic factors and older age at onset (130)], the development of CIPN is generally dose-dependent and thus influenced by different drug schedules and combinations, as well as route of administration. Traditionally, CIPN involves breast and colon cancer patients undergoing toxic doses of intravenous (IV) cisplatin and oxaliplatin. The cumulative toxicity mainly affects the DRG and their axons, causing a sensory neuronopathy or dying-back neuropathy; small fiber neuropathies can also ensue, while motor, autonomic, and cranial involvement is less common.

Most patients develop CIPN in the first three or four cycles of treatment, with gradual progression of symptoms and subsequent stabilization at or soon after treatment completion. Patients typically display length-dependent symmetric sensory loss (sometimes even leading to ataxia) with prominent acral sensory symptoms, pain episodes, and reduced dexterity. A notable exception is platinum-associated neuropathy, as oxaliplatin may also cause distinctive acute, transient, cold-induced dysesthesias after the first infusions, while both oxaliplatin and cisplatin neurotoxicity usually worsens in the months following the end of chemotherapy (i.e., the coasting phenomenon). CIPN then slowly improves with time, sometimes leaving a chronic pain syndrome with dysesthesia, hyperalgesia, tactile and thermal allodynia, and spontaneous pain. Acute pain syndrome related to nerve pathology has also been reported with paclitaxel (131). New anti-microtubule drugs can induce axonal, mainly sensory polyneuropathy, as can older taxanes [e.g., eribulin and ixabepilone (132)].

Recently, advancements in more selective targeted therapies and newer agents have improved chemotherapy tolerability and overall cancer survival but have not translated into a reduction of CIPN cases. Besides having to differentiate CIPN from other conditions that do not dictate premature treatment discontinuation, the advent of biological agents, known to have more idiosyncratic and off-target side-effects, has further added to the neurologist's diagnostic conundrum. Indeed, CIPN pathogenesis largely remains to be defined and is still one of the most common dose-limiting complications of antitumor treatment, considering the growing number of cancer survivors who develop late drug-resistant chronic pain syndromes that may heavily impact on their quality of life.

### Targeted Therapies

Tumor-specific antibodies cause similar, unexpected nerve injury ranging from mild, predominantly sensory neuropathies (i.e., polatuzumab vedotin and enfortumab vedotin) to potentially severe motor neuropathy, as seen in lymphoma patients undergoing brentuzumab (133, 134); these generally improve slowly upon discontinuation. Neuropathies have also been reported in high numbers during adotrastuzumab emtansine treatment (135). Regarding the latest drugs, such as neurotrophic-tyrosine-receptor-kinase gene and anaplastic-lymphoma-kinase inhibitors, neuropathy has been described with both larotrectinib [including grade III and IV reactions within 3 months of treatment (136)] and lorlatinib (137), respectively.

### Immune Checkpoint Inhibitors

Immune checkpoint inhibitors (ICIs) are monoclonal antibodies directed against specific molecules on activated immune cells whose role is to maintain self-tolerance and prevent autoimmunity, thus enhancing anti-tumor immunity. Targets include programmed death-1 receptor (nivolumab, pembrolizumab, cemiplimab), its ligand (atezolizumab, avelumab, durvalumab), and cytotoxic T lymphocyte antigen-4 (ipilimumab). Alongside, ICIs may cause off-target toxicities called immune-related adverse events (irAEs) (138, 139). Neurological irAEs represent a minor proportion, with an estimated incidence of 1–3%, more common after combination checkpoint blockade (140, 141). Nevertheless, they are clinically relevant complications with high-grade toxicity, carrying significant morbidity and mortality (142), and thus requiring prompt identification. Immune-related peripheral nerve disorders induced by ICI are highly heterogeneous (8, 9). Cranial neuropathies and polyradiculoneuropathies have emerged as the most common phenotypes (9). Cranial neuropathies may be isolated or associated with other neurological manifestations, frequently involving facial and abducens nerves, although any CN may be affected (143) Acute and chronic polyradiculoneuropathies mostly include GBS or CIDP and usually develop within the first three cycles of therapy (9, 144). Unlike classical GBS, cerebrospinal fluid analysis often reveals lymphocytic pleocytosis besides hyperproteinorrachia (142) and benefits from steroid administration. Other reported ICI-related neuropathies are mononeuritis multiplex/vasculitic neuropathy (8, 9), small fiber/autonomic neuropathy (145), sensory neuronopathies, and neuralgic amyotrophy (146, 147).

Despite these findings, ICIs are generally considered safer than conventional chemotherapy for the peripheral nerve, and ICI-induced neuropathies are more likely to have an acute/subacute and non-length-dependent presentation (9, 146).

According to current guidelines (148), management requires ICI discontinuation and high-dose IV steroid administration, followed by slow steroid tapering to avoid relapses related to the long-lasting half-life of ICIs (146). Up to 50% of cases (149) could be resistant to steroid therapy and, due to their severity, require IV immunoglobin (IG), plasma exchange, or immunosuppressants.

### CAR T Cell Therapy

Chimeric antigen receptor T-cell therapies have become standard treatments of relapsed or refractory hematological malignancies. Acute neurotoxicity is well-documented and moderate-to-severe neurological events are estimated to occur in 20–30% of patients, with a median time of onset of 5–6 days after infusion (150–152). Neurological manifestations are associated with cytokine release syndrome, referred to as “immune effector cell-associated neurotoxicity syndrome,” which identifies a distinctive encephalopathy manifesting with stereotypic evolution of a specific set of symptoms (152). Very little is known about potential long-term adverse events; however, a case of peripheral neuropathy has been described as a late complication (153).

## Therapy

The general therapeutic strategy for PNS is based on the assumption that the detection of cancer and its removal can improve the neurological syndrome. This is seldom obtained in PN associated with onconeural antibodies. Numerous attempts have been published for the treatment of SSN. A systematic review in 2012 concluded that only class IV evidence was available for the effect of IVIG, plasma-exchange, steroids, or immune-suppressive chemotherapy (53). Most individual recommendations suggest immune-modulating therapies can stop the progression of the PN. Given the severe debilitating outcomes in patients with SSN, these interventions are justified but should be performed very early (e.g., 2 months) after neurological symptoms onset (154). On the other hand, diseases associated with NSAbs are usually responsive to immunotherapy. This also seems to be confirmed for peripheral involvement in syndromes with anti-LGI1 anti-CASPR2 antibodies (47). Steroids are often the first option, followed by or associated with IVIG and plasma exchange. Therapies with anti-CD20 treatment (e.g., rituximab), or immunosuppressors like cyclophosphamide, have less supporting evidence than do other NSAbs associated diseases (e.g., NMDAR encephalitis) (155). Nevertheless, these options can be considered in non-responsive cases and their efficacy has been reported (44, 78, 156).

The smaller group of acute immune-mediated neuropathies, as GBS, CIDP, and vasculitis, usually respond to conventional immune therapies, approved for the non-neoplastic entity of the given neuropathy. Paraproteinemic neuropathies and AL amyloidosis are also treatable to some extent, according to current hematological guidelines.

The appearance of PN has implications not only for cancer and cancer therapy but also significantly impacts patient performance, quality of life, and disability. Symptomatic therapy is therefore a cardinal feature of treatment. Pain control is a key goal. This can be achieved by antidepressants (tricyclic antidepressants and serotonin-noradrenaline reuptake inhibitors) and by GABA-mimetic drugs (first-line therapy). Second- and third-line drugs for neuropathic pain include topical lidocaine and opioids (157). Neuromyotonia can respond to antiepileptics as carbamazepine, phenytoin, lamotrigine, and sodium valproate (158).

Cancer rehabilitation is an important initiative to promote specific therapies for patients affected by neurological conditions in cancer. Therapy effects and, due to increased long-term survival, persisting effects need to be specifically treated. The rehabilitation of neuropathies is generally also heterogeneous, depending on the focus of deficit and disability. The reference level could be at best adapted and copied from the rehabilitation of cancer patients with CIPN, which bears similarities with the most common phenotype.

## Conclusion

In summary, since the 2004 classification, several peripheral manifestations have been described, mainly with new antibodies, in patients affected by cancer. Whereas novel intracellular antibodies need more robust evidence to become relevant in clinical practice, the true novelty has been the discovery of NSAbs in diseases with prominent, or coexisting, peripheral involvement, which can be paraneoplastic.

Circulating autoantibodies are commonly considered a valuable tool for cancer diagnosis and are frequently requested in clinical practice for patients with unclear neurological peripheral symptoms. However, proper adherence to the use of biomarkers is critical in translating recommendations into clinical practice. At present, circulating classical onconeural antibodies and only a few NSAbs are considered a valuable tool for cancer diagnosis for patients with paraneoplastic neuropathy.

Finally, new cancer therapies seem to evoke immune-mediated neurological syndromes, which may mimic PN, but appear at different times during treatment. In the coming years, the study of the mechanisms and effects of these therapies will provide new insights into the relationship between cancer, immunity and the nervous system.

## Author Contributions

WG, MZ, AG, and BG conceived and drafted the review. VP and FB contributed to draft the review. WG and BG revised the manuscript for important intellectual content. All authors contributed to the article and approved the submitted version.

## Conflict of Interest

The authors declare that the research was conducted in the absence of any commercial or financial relationships that could be construed as a potential conflict of interest.

## Publisher's Note

All claims expressed in this article are solely those of the authors and do not necessarily represent those of their affiliated organizations, or those of the publisher, the editors and the reviewers. Any product that may be evaluated in this article, or claim that may be made by its manufacturer, is not guaranteed or endorsed by the publisher.
